# COVID-19, circadian rhythms and sleep: from virology to chronobiology

**DOI:** 10.1098/rsfs.2021.0043

**Published:** 2021-10-12

**Authors:** Zulian Liu, Sharlene Ting, Xiaodong Zhuang

**Affiliations:** ^1^ Nuffield Department of Population Health, University of Oxford, Oxford, UK; ^2^ Nuffield Department of Medicine, University of Oxford, Oxford, UK; ^3^ National Institute for Health and Care Excellence, UK

**Keywords:** SARS-CoV-2, coronavirus, dexamethasone, healthcare workers, vaccine

## Abstract

Various aspects of our physiology and immune response to pathogens are under 24 h circadian control and its role in clinical and research practice is becoming increasingly recognized. Severe acute respiratory syndrome coronavirus-2, the causative agent of Coronavirus disease 2019 (COVID-19) has affected millions of people to date. Cross-disciplinary approaches and collaborative efforts have led to an unprecedented speed in developing novel therapies and vaccines to tackle the COVID-19 pandemic. Circadian misalignment and sleep disruption have a profound impact on immune function and subsequently on the ability of individuals to combat infections. This review summarizes the evidence on the interplay between circadian biology, sleep and COVID-19 with the aim to identify areas of translational potentials that may inform diagnostic and therapeutic strategies in this pandemic.

## 1. Introduction

Severe acute respiratory syndrome coronavirus-2 (SARS-CoV-2), the causative agent of Coronavirus disease 2019 (COVID-19), has affected millions of people to date. Accumulating evidence suggests that the circadian clock can regulate the strength of immune responses to pathogenic insults and the susceptibility of organisms to viral infection [1]. The potential interaction of SARS-CoV-2 virology and pathology, and the host circadian clock has been proposed in several hypothetical and review articles [2–4].

The circadian clock is an internal biological timekeeper that drives 24 h rhythms of our behaviour and physiology, including sleep–wake and feeding–fasting cycles, hormone production and immune system. This built-in biological clock is evolutionarily conserved and exists in nearly all organisms, allowing the host to anticipate and respond to daily environmental changes. The master clock or the principal ‘pacemaker’ is located in the suprachiasmatic nucleus of the hypothalamus in the mammalian brain which links to a network of peripheral clocks in virtually every tissue and organ [5].

The circadian system has a central role in the sleep–wake cycle regulation, the most obvious 24 h rhythm in humans. In recent years, evidence has emerged highlighting the importance of sleep as a prominent regulator of innate and adaptive immunity [6]. Sleep pattern and quality can impact on immune response against viral, bacterial, and parasitic pathogens and reciprocally, infections may alter sleep patterns.

Viruses are obligate parasites that rely on host cell synthesis machinery to replicate. A major advancement in the field of chronobiology is the realization that host susceptibility to an infectious agent is not only dependent on the viral inoculum size, transmission route and length of exposure, but on the time of day when the pathogen is encountered. Multiple studies have demonstrated that many viruses are directly or indirectly influenced by the circadian-regulated pathways [7–10].

This review focuses on evidence-based findings to support the interplay between circadian rhythm, sleep and COVID-19 in order to explore how knowledge of chronobiology might be applied to mitigate propagation and severity of disease induced by SARS-CoV-2 infection.

## 2. Viral shedding may be dependent on the time of day

With increasing interest in the interplay between viruses and the host circadian clock, hypotheses have been proposed that the time of day regulates the host control of SARS-CoV-2 replication through circadian-regulated host susceptibility and immunity [11]. McNaughton *et al*. examined the results of more than 30 000 standard polymerase chain reaction tests of nasopharyngeal swab samples and showed that the test results were time-of-day dependent. A twofold variation was observed in the proportion of positive results across a 24 h period with a peak of positivity in the early afternoon (around 14.00). This suggests that the test results were most likely to be positive (less likely to be false negative) in the early afternoon compared with other times of day [12]. This observation is likely to be attributed to a combination of the circadian-regulated immune system and host pathways essential for SARS-CoV-2 replication, resulting in a fluctuation of viral shedding throughout the day. Consistently, a recent study in influenza using a mouse model showed that symptoms and viral shedding may vary across the 24 h period due to the circadian regulation of the immune system [13].

## 3. The molecular clock and severe acute respiratory syndrome coronavirus-2 infection

While further evidence on monitoring viral loads at different times of the day is required to confirm McNaughton's observation, Zhuang *et al*. demonstrated a role for circadian pathways in modulating the susceptibility of lung epithelial cells to SARS-CoV-2 infection [14]. In this study, the loss of the key circadian transcription activator BMAL1 resulted in a reduced expression of the major viral receptor, angiotensin-converting enzyme 2 (ACE2) and viral entry in lung epithelial cells. Nevertheless, since factors and mechanisms involved in SARS-CoV-2 entry are still being identified [15–17], and there is an extensive range of genes/pathways regulated by BMAL1, it is likely that additional circadian-regulated factors might contribute to SARS-CoV-2 entry. The same study further showed that silencing or pharmacological inhibition of BMAL1 induced a wide range of interferon-stimulated genes which possess a broad activity against many viruses [18], suggesting that targeting host clock proteins may be a possible approach to limiting not only SARS-CoV-2 but other viral infection. Consistently, Ray *et al*. found that 30% of the identified host factors interacting with SARS-CoV-2 showed circadian oscillation [4,19], highlighting the urgent need to increase our understanding of how the circadian clock influences SARS-CoV-2 infection.

## 4. Time of vaccine matters

Vaccine development against COVID-19 has been at an unprecedented speed. Several vaccines based on mRNA, vector and viral protein subunits have been developed and proven effective to elicit an appropriate immune response [20]. However, the ability of these vaccines to reduce the incidence of COVID-19 varies considerably [21,22]. While factors involving the emerging SARS-CoV-2 variants and the host age and health status are likely to contribute to the efficacy of these vaccines, the timing of vaccination has also been previously demonstrated to influence vaccine efficacy [23–25]. Phillips *et al*. [24] reported that vaccination in the morning induced greater antibody responses to both hepatitis A and influenza vaccines in man. A more recent larger randomized trial examined the impact of time of day on the antibody response to the annual influenza vaccination in the elderly and showed that morning vaccination (9.00–11.00) markedly increased viral-specific antibody responses compared with afternoon vaccination (15.00–17.00) [25]. These findings suggest that modulating the time of vaccination may provide a simple and practical measure to enhance vaccine efficacy, and that administering COVID-19 vaccines in the morning may result in greater protection. Further, the timing of vaccinations should be considered when evaluating efficacy of COVID-19 vaccine trials.

## 5. The clock synchronizer—dexamethasone and Coronavirus disease 2019

Glucocorticoid (GC) is a stress hormone released from the adrenal glands, which regulates metabolic processes, immune function and circadian physiology [26]. GC has been used clinically to treat auto-inflammatory diseases such as allergies, arthritis and asthma [27]. GC treatment has also been evaluated against respiratory viral infections including severe acute respiratory syndrome [28], Middle East respiratory syndrome [29] and other influenza-associated pneumonia [30]. Importantly, a synthetic GC, dexamethasone, has been used to treat critically ill patients with COVID-19, decreasing the death rate by about one-third [31]. In addition to its known immune-suppressive action, dexamethasone is widely used to synchronize the circadian clock in tissue cultures in laboratory conditions, further supporting the theory that clock synchrony may be beneficial in fighting infections. An interesting question remains as to whether there is an optimal time to deliver dexamethasone. Since clinical studies reported that night-time GC administration is more effective in preventing autoimmune flares by reducing pro-inflammatory cytokines during the night, it is plausible that night-time GC delivery may be more effective in reducing the risk of severe immuno-pathological consequences, such as cytokine storm, in COVID-19 patients.

## 6. The impact of Coronavirus disease 2019 on sleep

The COVID-19 pandemic has posed a significant challenge across multiple sectors from governments, healthcare systems, educational institutions and the general public. Infection outbreaks in combination with social restrictions to limit the viral spread have been associated with a rise in mental health issues, psychologic distress and impaired sleep quality, especially among healthcare workers [32,33]. Wang and colleagues studied the sleep disturbance and psychological profiles of medical and non-medical staff during the early outbreak of COVID-19 in Hubei Province, China in 2020. They found that 61.6% of healthcare workers reported sleep problems, and the prevalence of sleep disorders was higher among frontline healthcare workers compared to non-frontline and non-medical staff [34].

Disruptions to normal daily routines due to lockdowns during the pandemic period, such as confinement at home, can disrupt circadian rhythms that impact on sleep patterns in the general population. A cross-occupational survey in May 2020 reported a shift to later bedtime and waking time, with a reduction in night-time sleep and an increase in day-time napping compared to the pre-lockdown period [35]. In line with this observation, Innocenti *et al*. [36] reported a worsening trend of sleep patterns among general populations in Italy during the pandemic.

Alimoradi *et al*. [37] reported that among people with sleeping problems during the COVID-19 pandemic, 31% were healthcare professionals, 18% comprised the general population and 57% were COVID-19 patients. Importantly, a recent meta-analysis of 54 231 participants showed a dramatic increase in sleep problems in COVID-19 patients (74.8%) compared with the global pooled prevalence (35.7%) [38]. These studies highlighted sleep disruption as a common feature of infected individuals, who may be more prone to sleep-related illness.

While early symptoms in the acute infection phase such as coughs, breathing issues and anxiety could affect sleep, Huang *et al*. [39] reported that at six months after acute infection, patients with COVID-19 still experienced fatigue or muscle weakness, sleep difficulties and anxiety or depression [39]. Whether the virus perturbs circadian rhythm and affects sleep behaviour remains to be explored. Although COVID-19 is primarily considered a respiratory disease, SARS-CoV-2 affects multiple organ systems including the central nervous system. Song *et al*. using human brain organoids and mice overexpressing human ACE2, demonstrated that SARS-CoV-2 is capable of infecting neurons. Further, SARS-CoV-2 was detected in cortical neurons of the brain during autopsies of COVID-19 patients [40]. It is tempting to speculate that SARS-CoV-2 infection of neurons may lead to the perturbation of circadian rhythm and sleep in COVID-19 patients.

## 7. Sleep disruption as a risk factor for Coronavirus disease 2019

Sleep disruption has been associated with an increased risk of infectious diseases [41]. Kim *et al*. [42] recently reported that in the high-risk population of healthcare workers, sleeping for 1 h longer at night was associated with a 12% lower risk of COVID-19, whereas severe sleep problems were associated with an 88% greater risk of COVID-19. This finding suggests that severe sleep problems may be a potential risk factor for COVID-19 among healthcare workers, highlighting the urgent need to maximize the well-being of healthcare workers during the pandemic.

## 8. Shift work and Coronavirus disease 2019 healthcare workers

The term shift work often refers to any work scheduled outside the hours between 7.00 and 17.00 which include evening, night and early morning shifts with permanent or irregular schedules. Shift work is becoming more common at a global scale, and it is estimated that 25% of the workforce in the UK participate in some form of shift work. The adverse consequence of shift work has been associated with obesity, diabetes, cancer and increased risk for viral infections [43]. While the underlying mechanisms are still to be defined, sleep disruption and circadian misalignment are thought to be major contributors [44]. Healthcare workers are subject to chronic sleep restriction and shift work [45,46], and the intensity of which was even greater during the COVID-19 pandemic.

Several studies have investigated the association between shift work and COVID-19 status. Maidstone *et al*. [47] analysed data from the UK Biobank with more than 280 000 participants and found that shift workers treated in hospital were up to three times more likely to be COVID-19 positive compared with other in-hospital patients. Although the cause of this association is still unknown, it is plausible that the circadian clock controlling immune response may be impaired in shift workers, which resulted in the higher susceptibility to viral infection, in line with a previous study showing that shift workers were more susceptible to respiratory infections [48]. Consistently, Fatima *et al*. [49] reported that night shift workers had a 1.85-fold higher risk of contracting COVID-19 infection. Further, Rizza *et al*. [50] reported that, of people working on a rotating-night shift, those with a BMI > 30 had a greater risk of contracting COVID-19, suggesting that obesity is an additional risk factor for COVID-19.

## 9. Improved sleep in young populations

Adolescents typically show a delayed sleep pattern [51] which is commonly associated with sleep restriction and poor school performance [52]. As such, there have been suggestions to delay school start times to help improve the sleep quality and duration of adolescents [53]. The COVID-19 pandemic has resulted in school closure and promoted home-based online learning, which in turn may provide adolescents with the flexibility to align their sleep preferences and study schedules.

Genta *et al*. found that during the COVID-19 pandemic, high school students aged 15–16 years delayed bed and wake-up times by 1.5 and 2 h, respectively, and shifted their chronotype towards the evening. Improved sleep duration and quality was observed among those who had a shorter sleep duration pre-pandemic [54]. A report on university students aged 20–24 also suggests a positive impact of the lockdown on sleep behaviours [55]. Wright *et al*. investigated sleep behaviours prior to and during the home-learning period in university students and found an improved regularity of sleep timing and that time in bed at night devoted to sleep increased by approximately 30 min. These beneficial effects are also observed in participants with a wider age range (18–39 years), who showed a 1 h delay in wake-up times during the COVID-19 lockdown. Moreover, social jetlag, that is, the time difference between the midpoint of sleep on workdays and on free days—a consequence of the discrepancy between an individual's biological rhythm and the social clock [56]—decreased by nearly 1 h [57]. The improved sleep patterns are likely due to the late chronotype of the younger population and increased flexibility of social schedules which facilitated adaptation of sleep and work hours to the internal circadian rhythm.

## 10. Better sleep and vaccine efficacy

Given the prominent role of sleep in regulating the immune system [6], it is unsurprising that it also impacts on vaccine efficacy. An early study examined the effect of sleep deprivation on the immune response to an influenza vaccine and demonstrated that the IgG antibody titres from individuals with sleep deprivation at the time of vaccination were less than half of those with normal sleep times [58]. This finding was further supported by a recent trivalent influenza vaccine study, which showed that shorter sleep on the days preceding and after the vaccination was associated with a lower immune response even four months after the initial vaccination [59]. In a hepatitis A vaccine study examining the effects of sleep or being awake in the night following vaccination, participants who had slept showed a doubled frequency of Ag-specific T helper cells and increased antigen-specific IgG1 antibodies [60]. These findings suggest that sleep may promote immune responses to vaccination and may even act as an adjuvant to enhance vaccination efficacy. Although the impact of sleep on the immune response to COVID-19 vaccines is still to be determined, it is plausible, based on the lessons learnt from vaccines against other viral pathogens, that adequate sleep is likely to be beneficial for inducing a robust immune response against COVID-19.

## 11. Conclusion

This review aims to increase our appreciation of the role of circadian biology in SARS-CoV-2 replicative cycle and raise awareness of sleep quality in COVID-19 patients and healthcare workers during the COVID-19 pandemic. Understanding the factors underlying sleep impairment could inform public health measures with the goal to improve sleep quality and possibly vaccine efficacy. We advocate collecting ‘time stamps', that is, data on the time that clinical samples are collected and when treatments, including vaccinations are administered. This information could help to reshape a more efficient public health strategy to achieve an optimal clinical outcome.

## Data accessibility

This article has no additional data.
